# The effect of spreader graft use on voice in rhinoplasty cases: a prospective randomized controlled study

**DOI:** 10.1016/j.bjorl.2026.101847

**Published:** 2026-06-16

**Authors:** Burak Ulkumen, Sabri Mutlu, Onur Celik

**Affiliations:** Manisa Celal Bayar University, Faculty of Medicine, Department of Otorhinolaryngology, Manisa, Turkey

**Keywords:** Rhinoplasty, Structural, Voice analyses, Spreader graft

## Abstract

**Objective:**

This study aimed to evaluate the impact of spreader graft placement during cosmetic rhinoplasty on both objective acoustic measures and subjective voice quality.

**Methods:**

In this prospective, double-blind, controlled study, volunteers who underwent open structural rhinoplasty by the same surgeon between September 2022 and January 2025 were included. Participants were divided into two groups: Group A (with spreader grafts) and Group B (without spreader grafts). Voice recordings were obtained preoperatively and three months postoperatively in a dedicated voice and speech laboratory. All participants provided informed consent and completed the Turkish version of the Voice Handicap Index-10 (VHI-10) before and after surgery. Objective voice analysis was conducted using the Computerized Speech Lab (CSL) system and Multi-Dimensional Voice Program (MDVP) software (Kay Elemetrics Corp., Lincoln Park, NJ, USA). Acoustic parameters ‒ fundamental frequency (f0), jitter, jitter percentage, shimmer, shimmer percentage, and Noise-to-Harmonics Ratio (NHR) ‒ were measured from sustained /a/ phonations. Spectrographic analysis assessed formant frequencies (F1–F4) during production of nasal consonants (/m/, /n/) and the nasalized vowel /i/ in the word *mini*.

**Results:**

In Group A, significant postoperative differences were observed only in F3 and F4 formant values for /m/. In Group B, significant changes were detected in shimmer (dB), jitter, and formant values (F2, F3, F4 for /n/ and F3, F4 for /m/). Postoperative group comparison revealed significant differences in F4 for /m/, F3 for /n/, and F3 for the nasalized vowel /i/. Regarding subjective measures, VHI-10 scores significantly increased postoperatively only in Group B. Preoperative VHI-10 scores did not differ significantly between groups, while postoperative scores showed significant differences.

**Conclusion:**

Cosmetic rhinoplasty may affect voice parameters, particularly formants F3 and F4, essential components of the singer’s and actor’s formant cluster. Spreader grafts appear to preserve these frequencies, suggesting their importance in patients who professionally rely on voice quality.

**Level of evidence:**

According to the Oxford Centre for Evidence-Based Medicine 2011 Levels of Evidence, this study qualifies as Level 1b evidence, as it is a prospective, double-blind, randomized controlled trial evaluating the effectiveness of an intervention.

## Highlights

• Spreader grafts protect essential voice formants in aesthetic rhinoplasty cases.
• Significant acoustic deterioration observed in patients without spreader grafts.
• Rhinoplasty may alter F3–F4 formants, affecting singer’s and speaker’s clusters.
• Spreader grafts prevented postoperative rise in VHI-10 voice handicap scores.
• First prospective double-blind trial evaluating voice outcomes in rhinoplasty.

## Introduction

Nasal morphology significantly varies across different geographic regions and populations, necessitating distinct approaches in rhinoplasty surgery. Augmentation procedures are typically required among individuals of Southeast Asian and African descent, whereas reduction techniques are more frequently employed for populations from the Middle East and Northeast Asia, including Türkiye.^1^^,^^2^ Augmentation rhinoplasty typically has minimal impact on the internal nasal valve region and may even improve nasal patency. Conversely, substantial hump reduction commonly leads to a notable decrease in both the cross-sectional area and angle of the internal nasal valve.^3^ These anatomical alterations may consequently affect nasal airflow dynamics and critical voice parameters. One of the primary methods to mitigate such changes in structural rhinoplasty is the application of spreader grafts,^3^ which serve to prevent cosmetic deformities (e.g., inverted-V deformity, dorsal narrowing) as well as functional impairments (e.g., hyponasality, altered voice timbre, nasal obstruction).

Human voice production results from a complex interaction between the lungs, vocal cords, and the upper airway.^4^ The process begins with expiratory airflow generated by the lungs, which induces vocal cord vibrations through changes in transluminal pressure. This phenomenon, explained by the Bernoulli principle, generates the fundamental Frequency (F0) of the voice.^5^^,^^6^ In the subsequent phase, the final unique quality of the voice is shaped by the resonant properties of the sub-regions of the upper airway, including the pharynx, tongue, soft palate, nasal cavity, and paranasal sinuses. Surgical interventions involving these anatomical regions are expected to influence voice resonance characteristics. This assumption has been partially supported by previous studies.^7^, ^8^, ^9^, ^10^, ^11^, ^12^ However, the effects of various surgical interventions on the upper airway, specifically on formants (spectral peaks), which serve as measurable indicators of upper airway-related resonance characteristics in human voice, have not yet been fully elucidated.^13^^,^^14^ While these potential changes may not significantly impact overall quality of life, they can present performance challenges for professional voice users such as singers and actors.^14^ If the performed surgery is purely cosmetic, such as rhinoplasty, voice alterations may lead to serious medicolegal consequences. A review of the literature concerning medicolegal issues related to complications of rhinoplasty and informed consent reveals a lack of consideration for voice changes.^15^, ^16^, ^17^ Additionally, the importance of higher formants, particularly F3, F4, and F5, in the formation of the singer’s and actor’s formant clusters should not be overlooked.^18^^,^^19^ Narrowing of the nasal cross-sectional area following cosmetic rhinoplasty may affect these formant clusters, especially during the production of nasalized syllables, posing significant performance challenges for professional voice users.

Although several studies have investigated the effects of nasal surgery on human voice, no prospective controlled study has specifically examined the protective effect of spreader grafts on voice alterations in purely cosmetic rhinoplasty.^20^, ^21^, ^22^, ^23^, ^24^, ^25^, ^26^, ^27^, ^28^, ^29^ In this study, we aimed to determine whether the application of spreader grafts in cosmetic structural rhinoplasty could prevent adverse changes in human voice, particularly in the production of nasalized syllables.

## Methods

### Study design and sample selection

The study was designed as a prospective, controlled, double-blind trial, and ethical approval was obtained on September 21, 2022. Volunteer patients who underwent open technique rhinoplasty between September 2022 and January 2025 were included in the study.

A priori power analysis using G*Power 3.1.9.7 was conducted, assuming an effect size of 0.5 and power of 0.95. Required sample size was 45 for dependent and 176 for independent sample *t*-tests. Inclusion criterion was primary cosmetic rhinoplasty. Exclusion criteria included allergy history, prior nasal surgery, nasal obstruction, inferior turbinate hypertrophy, and significant septal deviation.

**Fig. 1:**
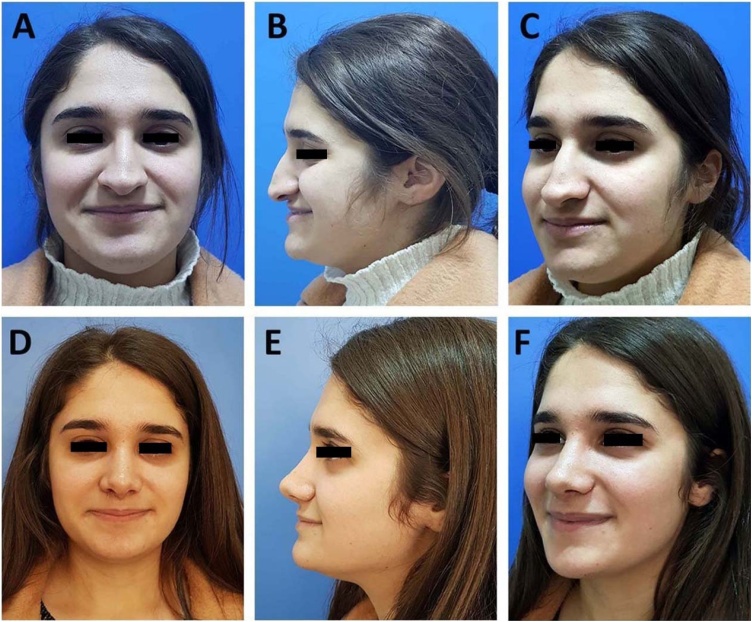
Preoperative (A, B, C) and postoperative (D, E, F) photographs of a case with bilateral spreader graft application, showing frontal, profile, and oblique views, respectively.

**Fig. 2:**
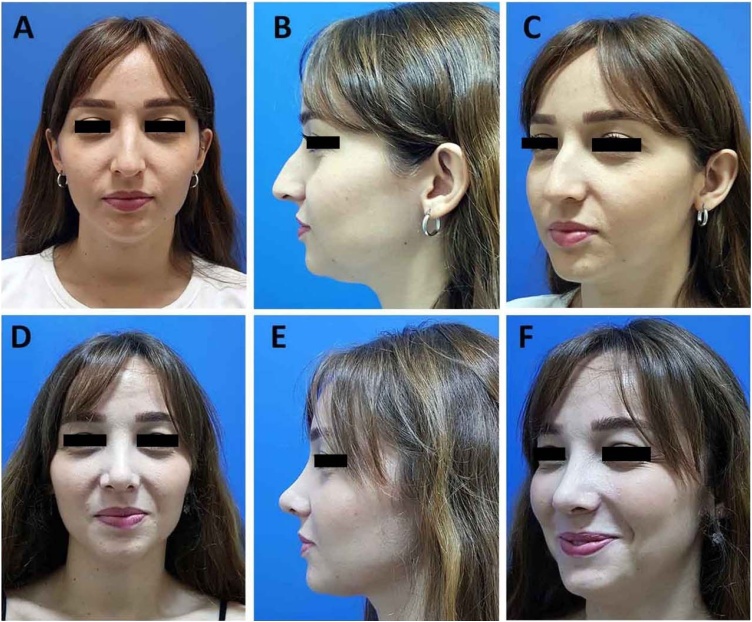
Preoperative (A, B, C) and postoperative (D, E, F) photographs of a case without spreader graft application, showing frontal, profile, and oblique views, respectively.

All patients were informed about the study design, surgical procedure, and participation, and provided written informed consent. They were not informed of their group allocation. Group allocation followed admission order; every other patient received a spreader graft, yielding an alternating 1:1 spreader versus non-spreader assignment. Due to the double-blind design, the researcher responsible for voice recordings was also blinded. Patients were then divided into two groups: those who received spreader grafts (Group A) and those who did not (Group B) (Fig. 1, Fig. 2).

### Surgical technique

All patients underwent structured primary rhinoplasty under general anaesthesia. Following sub-mucoperichondrial injection of a 1% lidocaine and 1:100,000 epinephrine mixture into both external and endonasal areas, a Goodman incision was performed. The lower lateral and upper lateral cartilages, along with the cartilaginous dorsum, were dissected in a supra-perichondrial plane, while the osseous dorsum was elevated in a subperiosteal plane.

Subsequently, the caudal border of the septum was exposed through the medial crura of the alar cartilages, and the quadrangular cartilage, maxillary crest, and vomer were elevated in a sub-mucoperichondrial plane using a #15 blade and a Freer elevator. If septal deviation was present, it was corrected using Blakesley forceps and an osteotome, depending on the location and type of deviation (bony/cartilaginous). Deviated bony or cartilaginous septal segments were excised following septotomy while ensuring the preservation of the L-strut. At the end of the procedure, excised but unused cartilage was crushed using a cartilage crusher and placed bilaterally between the mucoperichondrium inferior to the L-strut.

**Fig. 3:**
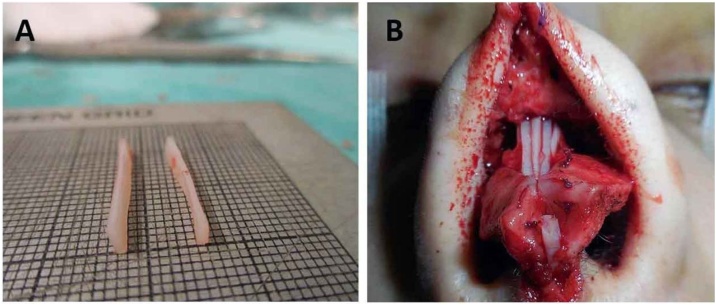
Bilateral spreader grafts prepared intraoperatively on a Sheen grid (A). Appearance of spreader grafts fixed to the dorsal border of the septal cartilage with horizontal mattress sutures (B).

Based on intraoperative evaluation, a spreader graft was fashioned from harvested septal cartilage, measuring 1–3 mm in thickness, 4–6 mm in width, and 20–35 mm in length (Fig. 3A). In all patients, the junction between the upper lateral cartilages and the septum was separated using an #11 blade. If an osseous hump was present, it was reduced using a 10–14 mm Rubin osteotome or bidirectional rasps (#2–8), depending on anatomical needs. Final osseous vault shape was achieved via lateral, transverse, median, and medial osteotomies as required. The cartilaginous dorsum was refined by resecting the dorsal borders of the upper lateral and quadrangular cartilages to reach the desired height.

Following randomization, spreader grafts were applied in Group A patients (Fig. 1). Grafts were placed bilaterally along the septal cartilage, aligned with the dorsal border in the sagittal plane, and secured with horizontal mattress sutures, ensuring the anterior edge did not extend beyond the caudal margin of the upper lateral cartilage (Fig. 3B). The upper lateral cartilages were then sutured to the grafts using 5‒0 round PDS sutures to complete dorsum reconstruction. After tip-plasty and placement of a columellar strut or septal extension graft, the incision was closed with 6−0 polypropylene sutures. The procedure was concluded with the application of a dorsal splint and placement of Doyle intranasal splints.

### Voice assessment

Voice recordings of the patients were obtained preoperatively and at the third postoperative month in our clinic's Voice and Speech Disorders Unit. Additionally, all patients signed an informed consent form regarding the study and completed the Turkish version of the VHI-10 questionnaire^4^^,^^30^^,^^31^ before and after surgery. This index consists of 10 subcategories that assess the impact of voice disorders on daily life. Scores range from 0 to 4 based on the frequency of the problem (0 = never, 1 = almost never, 2 = sometimes, 3 = almost always, 4 = always).

For voice analysis, the CSL (Computerized Speech Lab) system, developed by Kay Elemetrics Corporation, and the MDVP (Multi-Dimensional Voice Program) software were utilized. A Pentium 4 (3.00 GHz) processor computer, a Sound Blaster Live Value sound card, and an Audio-Technica AT2005 dynamic microphone were used for recording and analyzing voice data. Voice recordings were obtained after a 15-minute resting period, with patients sitting upright and maintaining a 15 cm distance from the microphone.^32^ They were instructed to sustain the /a/ vowel sound for 5 seconds and pronounce the word /mini/, which contains the nasal consonants /m/ and /n/, at a comfortable speaking pitch.

The recorded voice samples were analyzed using the MDVP acoustic analysis program. Acoustic parameters, including fundamental frequency (f0), jitter, jitter percentage, shimmer, shimmer percentage, and Noise-to-Harmonics Ratio (NHR), were calculated based on sustained /a/ vowel recordings for each patient. Spectrographic analysis was performed to determine the formant frequencies F1, F2, F3, and F4 from the nasal consonants /m/ and /n/ in the word /mini/ and the nasalized /i/ vowel following the nasal consonants.

### Statistical analysis

The distribution of the data was assessed using the Shapiro–Wilk test. Variables following a normal distribution were presented as mean (Standard Deviation [SD]), while non-normally distributed variables were reported as median (Interquartile Range [IQR]). For each group, preoperative and postoperative values of each parameter were compared using either a paired *t*-test or the Wilcoxon signed-rank test, depending on the results of the Shapiro–Wilk test. Independent sample *t*-tests were used to compare postoperative values between groups. Statistical significance was defined as p < 0.05. Statistical analyses were performed using the Statistical Package for the Social Sciences (SPSS) Version 21.0 (IBM Corp., Armonk, NY, USA).

## Results

### Descriptive statistics

A total of 125 female patients (mean age ± SD: 33.846 ± 11.261 years) and 65 male patients (mean age ± SD: 33.472 ± 9.101 years) were included in the study. The assessment of data distribution for cases in Groups A and B using the Shapiro-Wilk test is summarized in supplemental Table 1. When one or both of the paired preoperative and postoperative data sets within a group showed a non-normal distribution, the Wilcoxon signed-rank test was used; otherwise, a paired *t*-test was applied. For intergroup comparisons, the Mann–Whitney *U*-test was used when one or both postoperative data sets showed a non-normal distribution, whereas an independent sample *t*-test was used when both sets followed a normal distribution.

**Table 1:** Evaluation of the distribution of variables in cases with (Group A) and without (Group B) spreader graft application using the Shapiro–Wilk test.

Variables	p-value	
	(Group A)	(Group B)
Age	0.006^a^	0.000^a^
VHI^pre^	0.000^a^	0.000^a^
VHI^post^	0.000^a^	0.000^a^
F0^pre^	0.075	0.061
F0^post^	0.142	0.004
Jitta^pre^	0.000^a^	0.167
Jitta^post^	0.922	0.758
Jitt^pre^	0.000^a^	0.021
Jitt^post^	0.567	0.016^a^
ShdB^pre^	0.556	0.139
ShdB^post^	0.017^a^	0.303
Shim^pre^	0.348	0.021^a^
Shim^post^	0.027^a^	0.095
NHR^pre^	0.001^a^	0.108
NHR^post^	0.423^a^	0.416
F1 /m/^pre^	0.275	0.001^a^
F1 /m/^post^	0.358	0.003^a^
F2 /m/^pre^	0.774	0.295
F2 /m/^post^	0.019^a^	0.798
F3 /m/^pre^	0.000^a^	0.225
F3 /m/^post^	0.996	0.237
F4 /m/^pre^	0.856	0.216
F4 /m/^post^	0.922	0.516
F1 /n/^pre^	0.000^a^	0.002^a^
F1 /n/^post^	0.159	0.523
F2 /n/^pre^	0.265	0.056
F2 /n/^post^	0.230	0.403
F3 /n/^pre^	0.814	0.807
F3 /n/^post^	0.208	0.394
F4 /n/^pre^	0.039^a^	0.777
F4 /n/^post^	0.493	0.658
F1 /i/^pre^	0.505	0.493
F1 /i/^post^	0.213	0.868
F2 /i/^pre^	0.483	0.077
F2 /i/^post^	0.777	0.319
F3 /i/^pre^	0.000^a^	0.150
F3 /i/^post^	0.216	0.100
F4 /i/^pre^	0.558	0.670
F4 /i/^post^	0.324	0.669

Pre, Preoperative; Post, Postoperative.

a Variables showing abnormal distribution.

The gender distribution between the groups was evaluated using the Chi-square test, and no significant difference was found (p = 0.878). Similarly, no significant difference was observed in terms of age between Group A (33 [26–40], Median [IQR]) and Group B (32 [26–38], Median [IQR]) (p = 0.273).

### Comparative analysis of voice parameters

When comparing preoperative and postoperative voice parameters in Group A, significant changes were observed only in the F4 /m/ and F3 /m/ parameters (Table 2). In Group B, significant changes were noted in ShdB, F3 /m/, F4 /m/, F2 /n/, F3 /n/, F4 /n/, and jitter parameters (Table 3). When preoperative values of both groups were considered, no significant difference was found in any parameter except F2 /n/ (Table 4). However, when postoperative measurements of both groups were compared, statistically significant differences were observed in F4 /m/, F3 /n/, and F3 /i/ values (Table 5).

**Table 2:** Comparison of preoperative and postoperative voice analysis values in Group A.

Variables with normal distribution	Preoperative (mean ± SD)	Postoperative (mean ± SD)	p
F0	124.42 ± 45.36	127.70 ± 41.17	0.596
F1 /m/	3261.07 ± 657.90	3289.07 ± 678.86	0.764
F4 /m/	13405.77 ± 1254.39	13173.14 ± 1406.99	0.048^a^
F2 /n/	7067.31 ± 1228.19	6859.08 ± 121261	0.133
F3 /n/	103.94 ± 1268.23	10151.58 ± 1412.28	0.180
F1 /i/	3054.73 ± 1615.37	3433.76 ± 2412.41	0.208
F2 /i/	7393.55 ± 1419.10	7112.95 ± 2269.82	0.278
F4 /i/	14312.01 ± 1902.59	13896.33 ± 18.2642	0.066

Variables with abnormal distribution	Preoperative (Median [IQR])	Postoperative (Median [IQR])	p
Jitta	97.07 (18.31–202.27)	77.26 (44.26–10607)	0.112
Jitt	0.821 (0.134–1.900)	0.926 (0.514–1.332)	0.897
ShdB	0.285 (0.195‒0.400)	0.297 (0.153‒0.390)	0.945
Shim	3.448 (2.229–4.914)	3.643 (2.598–5.091)	0.474
NHR	0.132 (0.109‒0.151)	0.132 (0.109‒0.151)	0.604
F2 /m/	7205.81 (6031.83–8207.20)	7256.81 (6132.54–8294.26)	0.870
F3 /m/	10701.53 (-9860.03–11505.19)	10092.80 (9041.50–10809.42)	0.000^a^
F1 /n/	3453.06 (3002.77–3809.92)	3208.00 (2858–3731)	0.028
F4 /n/	12825 (12121.98–14068.52)	12806.98 (11712–13603)	0.156
F3 /i/	11144.43 (9951.87–12426.36)	10067.46 (8410.90–10944.05)	0.056
VHI-10	3 (0–11)	2 (0–9)	0.123

Pre, Preoperative; Post, Postoperative; IQR, Interquartile range.

a Variables with non-normal distribution.

**Table 3:** Comparison of preoperative and postoperative voice analysis values in Group B.

Variables with normal distribution	Preoperative (mean ± SD)	Postoperative (mean ± SD)	p
F0	123.34 ± 39.15	132.21 ± 35.11	0.245
Jitta	113.19 ± 178.64	85.12 ± 54.31	0.808
ShdB	0.29 ± 0.16	0.31 ± 0.17	0.040^a^
NHR	0.13 ± 0.03	0.12 ± 0.02	0.428
F2 /m/	6764.30 ± 1570.63	7028.54 ± 1140.08	0.536
F3 /m/	10509 ± 1422.93	9794.91 ± 1193.10	0.034^a^
F4 /m/	13387.59 ± 1409.94	12625.18 ± 1648.35	0.001^a^
F2 /n/	7525.03 ± 1152.76	6779.46 ± 1396 ± 90	0.000^a^
F3 /n/	10418.96 ± 1137.31	9537.29 ± 1113.35	0.034^a^
F4 /n/	13446.04 ± 1688.12	12798.91 ± 1278.46	0.003^a^
F1 /i/	3136.47 ± 1548.77	2859.17 ± 2683.63	0.816
F2 /i/	7483.33 ± 1377.07	6975.71 ± 2315.22	0.673
F3 /i/	11075.16 ± 1979.46	9879.79 ± 1678.83	0.970
F4 /i/	14719.63 ± 2150	13609.27 ± 1967.62	0.723

Variables with abnormal distribution	Preoperative (Median [IQR])	Postoperative (Median [IQR])	p
Jitt	1.192 (0.474–2.239)	0.866 (0.559–1.282)	0.017^a^
Shim	3.052 (2.172–4.381)	3.224 (2.250–4.556)	0.976
F1 /m/	3258.47 (2815.18–3584.72)	3325.41 (2792.79–3743.02)	0.125
F1 /n/	3271.27 (2816.38–3772.13)	3225.53 (2749.53–3693.74)	0.027^a^
VHI-10	2 (0–6)	6 (1–14)	0.000^a^

IQR, Interquartile Range; SD, Standard Deviation.

a Statistically significant difference between measurements.

**Table 4:** Comparison of preoperative voice analysis values between both groups.

Variables with normal distribution	Group A (mean ± SD)	Group B (mean ± SD)	p
F0	124.42 ± 45.37	123.35 ± 39.16	0.861
ShdB	0.29 ± 0.15	0.29.12 ± 0.16	0.947
F2/m/	7018.73 ± 1440.24	6764.31 ± 1570.63	0.246
F4/m/	13405.77 ± 1254.39	13387.59 ± 1409.95	0.925
F2 /n/	7067.31 ± 1228.19	7525.04 ± 1152,76	0.009^a^
F3 /n/	10394.53 ± 1268.24	10418.96 ± 1137.32	0.889
F1 /i/	3054.73 ± 2412.41	3136.47 ± 1548.77	0.722
F2 /i/	7393.56 ± 1419.11	7483.34 ± 1377.08	0.659
F4 /i/	14312.02 ± 1902.59	14719.63 ± 2150.51	0.168

Variables with abnormal distribution	Group A (Median [IQR])	Group B (Median [IQR])	p
Jitta	97.07 (18.31–202.28)	97.80 (17.64–235.16)	0.673
Jitt	0.297 (0.153‒0.390)	0.303 (0.197‒0.421)	0.056
Shim	3.448 (2.229–4914)	3.052 (2.172–4.381)	0.264
NHR	0.132 (0.109‒0.151)	0.132 (0.112‒0.154)	0.540
F1/m/	3272.06 (2861.38–3605.34)	3258.47 (2815.19–3584.72)	0.565
F3/m/	10701.53 (9860.03–11505.19)	10501.46 (9734.16–11240.03)	0.247
F1/n/	3453.06 (3002.78–3809.92)	3271.27 (2816.39–3772.13)	0291
F4/n/	12825.10 (12121.98–14068.52)	12892.49 (12095.34–14378.28)	0.401
F3/i/	11345.75 (10416.41–12484.32)	11120.54 (9557.38–12353.42)	0.085
VHI-10	3 (0–11)	6 (1–14)	0.080

IQR, Interquartile Range; SD, Standard Deviation.

a Statistically significant difference between measurements.

**Table 5:** Comparison of postoperative voice analysis values between both groups.

Variables with normal distribution	Group A (mean ± SD)	Group B (mean ± SD)	p
F0	127.70 ± 41.17	132.21 ± 35.11	0.418
Jitta	76.67 ± 51.11	85.12 ± 54.31	0.271
F3/m/	9999.51 ± 1236.01	9794.91 ± 1193.10	0.247
F4/m/	13173.14 ± 1406.99	12625.18 ± 1648.35	0.015^a^
F1 /n/	3267.97 ± 723.71	3176.49 ± 727.36	0.386
F2 /n/	6859.08 ± 121261	6779.46 ± 1396 ± 90	0.675
F3 /n/	10151.58 ± 1412.28	9537.29 ± 1113.35	0.001^a^
F4 /n/	12683.44 ± 1316.26	12798.91 ± 1278.46	0.540
F1 /i/	3433.76 ± 2412.41	2859.17 ± 2683.63	0.122
F2 /i/	7112.95 ± 2269.82	6975.71 ± 2315.22	0.680
F3 /i/	11024.10 ± 2027.78	9879.79 ± 1678.83	0.000^a^
F4 /i/	13896.33 ± 18.2642	13609.27 ± 1967.62	0.299

Variables with abnormal distribution	Group A (Median [IQR])	Group B (Median [IQR])	p
Jitt	0.926 (0.514–1.332)	0.866 (0.559–1.282)	0.980
ShdB	0.297 (0.153‒0.390)	0.303 (0.197‒0.421)	0.432
Shim	3.643 (2.598–5.091)	3.224 (2.250–4.556)	0.202
NHR	0.132 (0.109‒0.151)	0.129 (0.113‒0.114)	0.919
F1/m/	3272.23 (2884.53–3770.56)	3325.41 (2792.79–3743.02)	0.992
F2/m/	7256.81 (6132.54–8294.26)	7031.11 (6233.45–7887.67)	0.515
VHI-10	2 (0–9)	6 (1–14)	0.002^a^

IQR, Interquartile Range; SD, Standard Deviation.

a Statistically significant difference between measurements.

Postoperative VHI-10 scores showed no statistically significant change in Group A, whereas a significant increase was observed in Group B (Table 2, Table 3). While there was no significant difference between the preoperative VHI-10 scores of the two groups, a statistically significant difference was found between their postoperative VHI-10 scores (Table 4, Table 5).

The differences in the categorization of variables as normally or abnormally distributed in supplemental Table 2, Table 3, Table 4 are based on variations in the statistical method applied to the compared variables. For instance, while the F1 /m/ variable is categorized under normal distribution in supplemental Table 2, it appears under abnormal distribution in supplemental Table 3, Table 4, Table 5. This discrepancy arises because the preoperative and postoperative values of this variable in Group A exhibit a normal distribution, whereas in Group B, the preoperative and postoperative values show an abnormal distribution (Table 1).

## Discussion

In this study, we investigated the impact of spreader grafts in rhinoplasty on voice outcomes by comparing patients who underwent rhinoplasty with spreader grafts to those without. Our findings indicate that incorporating spreader grafts can produce notable differences in both acoustic (spectrographic) and subjective voice characteristics compared to rhinoplasty performed without spreader grafts. Spectrographic analysis, particularly of the nasalized word /mini/, revealed statistically significant variations in a greater number of acoustic parameters among patients without spreader grafts. Specifically, in cases lacking spreader grafts, significant changes were detected in fundamental voice parameters such as ShdB and jitter, as well as in the third (F3) and fourth (F4) formants of the consonants /m/ and /n/, and in the second formant (F2) of the consonant /n/. Conversely, patients who received spreader grafts showed significant acoustic changes exclusively in the third and fourth formants (F3 and F4) of the consonant /m/.

The production of the nasalized syllables in the word /mini/, especially its consonants /m/ and /n/, requires maintaining closure of the oral cavity, allowing sound to resonate through the sinonasal passages. It is well-documented in the literature that anatomical alterations within these regions can influence nasal resonance, thereby affecting voice perception.^4^ Our findings not only support this theory but also suggest that spreader grafts have a protective role in preserving certain objective voice parameters. Moreover, subjective patient perception, assessed via the VHI-10 score, demonstrated no significant postoperative changes among patients who received spreader grafts. In contrast, a significant increase in the VHI-10 scores was observed in patients without spreader grafts, further underscoring the beneficial effect of spreader grafts on patient-perceived voice outcomes.

The frequencies of the first (F1) and second (F2) formants correlate primarily with vowel identity, while higher formants such as F3 and F4 significantly influence the unique quality and timbre of an individual's voice.^33^ Variations in tongue position, soft palate elevation, and lip shape alter these frequencies, which is essential for accurate vowel perception. According to our study, the absence of significant postoperative changes in F1 and F2 in both patient groups indicates that vowel articulation was preserved. However, alterations in F3 and F4 frequencies and elevated VHI-10 scores in patients without spreader grafts suggest rhinoplasty can influence perceptions of hyponasality and individual voice timbre. This potential impact warrants particular attention, especially for professional voice users. The notable F3 and F4 changes in patients without spreader grafts, specifically during articulation of /m/ and /n/ consonants, are significant given their possible effect on the “singer’s formant cluster”, a convergence of the third, fourth, and occasionally fifth formants.^14^^,^^18^^,^^19^^,^^34^

The singer’s formant cluster is characterized by a spectral peak around 3000 Hz, created by deliberate modifications in vocal tract resonance, such as lowering the larynx, typically achieved through vocal training.^30^ This cluster allows a singer’s voice to project over orchestral accompaniment without extra vocal effort or strain, enhancing brightness and clarity. Similar resonant convergence, termed the “speaker’s formant cluster”, is also crucial in acting performances.^19^ Based on our results, the protective effect of spreader grafts on F3 and F4 parameters in structural rhinoplasty indicates their role in preserving the integrity of the singer’s and speaker’s formant clusters, particularly benefiting professional voice users such as singers and actors.

Another expected voice change following rhinologic surgery is altered nasality perception. Thus, the protective effect of spreader grafts on specific fundamental and spectrographic voice parameters noted in our study may also hold significance regarding nasality perception. However, literature suggests that nasality perception might be more closely associated with reductions in formant amplitude rather than frequency changes.^20^ Consequently, the lack of amplitude analysis in formant assessments constitutes a limitation of our study. Additionally, interindividual variations in nasal deformities and cross-sectional nasal areas introduce inherent variability to the results. Nevertheless, given our substantial sample size and the implementation of double-blind randomization, we believe we have effectively minimized this potential bias. Because our primary focus was voice outcomes, we did not prospectively or statistically compare aesthetic satisfaction between groups, so we cannot link the favorable voice profile of the spreader graft group to cosmetic results. No graft-related cosmetic complaints were noted, but this was not evaluated with validated aesthetic measures, despite their expected structural aesthetic benefits.

The importance of the upper respiratory tract, particularly the nasal cavity and paranasal sinuses, in vocal resonance is widely acknowledged.^10^^,^^13^^,^^23^^,^^35^, ^36^, ^37^, ^38^, ^39^ Rhinologic surgeries are expected to significantly affect vocal resonance characteristics, especially during the production of nasalized syllables, which was the primary focus of our investigation. Previous studies, including our own, have demonstrated that septoplasty and turbinate surgery ‒ other rhinological procedures ‒ particularly influence the F3 and F4 formants.

Relying solely on subjective and objective voice assessments may not fully capture nasality-related voice changes resulting from rhinologic surgery and alterations in nasal cavity patency. Therefore, spectrographic analysis of formants during the production of nasalized vowels is essential.^22^^,^^21^ In a previous study, we conducted spectrographic analyses to evaluate formant frequencies in cases undergoing septoplasty and turbinoplasty ‒ two commonly performed rhinologic procedures ‒ and identified statistically significant changes.^14^

Studies evaluating the effects of different rhinologic surgeries on voice using spectrographic analysis date back to 1997.^9^ However, these studies predominantly focused on functional nasal pathologies such as nasal polyposis, septoplasty, and endoscopic sinus surgery, where improvements in nasal patency typically enhance both voice quality and nasality.^22^^,^^26^, ^27^, ^28^, ^29^ Conversely, primary structural rhinoplasty performed solely for cosmetic purposes involves patients without preoperative nasal obstruction or hyponasality, and thus the primary objective should be preserving existing voice quality. This distinction makes cosmetic rhinoplasty uniquely relevant regarding its potential postoperative voice alterations.^14^

Studies specifically examining rhinoplasty’s impact on voice remain limited.^25^^,^^40^, ^41^, ^42^, ^43^ Existing research primarily comprises cohort studies, case series, and cross-sectional studies with small sample sizes. To date, no prospective controlled study assessing rhinoplasty’s effect on voice has been reported. Regarding spreader graft use, only a cohort study by Celik et al., involving 20 patients, exists^44^ Celik et al. reported improved VHI-10 scores among patients receiving spreader grafts without significant changes in fundamental and spectrographic voice parameters, suggesting a protective effect. However, due to the lack of a control group, the evidence remains limited. Given our prospective, double-blind, controlled trial design, our study represents a unique and significant contribution to the literature.

## Conclusions

This study uniquely contributes to the literature as the first prospective, double-blind, controlled trial assessing both subjective (VHI-10) and objective (acoustic/spectrographic) voice outcomes following rhinoplasty with or without spreader grafts. Our findings highlight the importance of preserving sinonasal anatomical integrity to maintain optimal vocal resonance, emphasizing the protective role of spreader grafts particularly for professional voice users. This study not only bridges a significant gap in the current rhinologic literature but also provides robust evidence supporting the integration of spreader grafts into rhinoplasty procedures, especially in patients for whom voice quality and resonance are critically important.

## Patient consent statement

Written informed consent was obtained from every participant. A sample of the written patient consent form has been approved by the relevant ethics committee and can be obtained from the ethics committee upon request.

## Funding

No external funding was used for this study. The surgical equipment and voice analysis software and equipment used for the patients included in the study were already available in our clinic, and no additional budget was required.

## Ethics approval statement

It was approved by the Manisa Celal Bayar University Faculty of Medicine Health Sciences Ethics Committee (protocol number: 20.478.486/1515).

## Data availability statement

The authors declare that all data are available in repository.

## Declaration of competing interest

The authors declare no conflicts of interest.
